# Borrowing Trouble

**Published:** 1875-06

**Authors:** 


Borrowing Trouble.—Never borrow trouble; it will come soon enough; you have no need to shiver in the chilling shadow of a far-off woe. A gloomy habit of heart and mind is wrong, because it unfits one for duty. Our dispositions, like plants, need sunshine. Expectancy of repulse is the cause of many religious and secular failures. Fear of bankruptcy has uptorn many a fine business, and sent the men dodging among the note shavers. Fear of slander and abuse has often invited all the long-beaked vultures of backbiting. Many of the misfortunes of life, like hyenas, flee if you courageously meet them. So, when trouble comes, bear it bravely, but do not weaken your powers of endurance by anticipating it.

---

There is nothing so beneficially educating to a young man as the companionship of sisters. They laugh him out of those little awkwardnesses of manner which otherwise might become habitual. They refine him unconsciously in all matters of taste and politeness. They nip the little buds of puppyism which, under other circumstances, might flaunt their flowers before less partial eyes. When brothers refuse to accompany their sisters, in order to dance attendance upon other young ladies, let them remember who made them presentable and agreeable to the other young ladies.

---

Better a little furniture than an empty house.

